# Interleukin-4 inhibition of interleukin-1-induced expression of matrix metalloproteinase-3 (MMP-3) is independent of lipoxygenase and PPARγ activation in human gingival fibroblasts

**DOI:** 10.1186/1471-2199-8-12

**Published:** 2007-02-23

**Authors:** Denise Stewart, Masoud Javadi, Mariah Chambers, Chad Gunsolly, Grzegorz Gorski, Ruth C Borghaei

**Affiliations:** 1 Department of Biochemistry and Molecular Biology, Philadelphia College of Osteopathic Medicine, Philadelphia, PA, USA

## Abstract

**Background:**

Interleukin 4 (IL-4) has been shown to suppress interleukin-1 (IL-1) induced expression of matrix metalloproteinase-3 (MMP-3) in human synovial and gingival fibroblasts, but the mechanism of suppression has not been determined. Activators of peroxisome proliferator-activated receptor-γ (PPARγ) have been shown to inhibit cytokine induced expression of MMPs in other cell types, and IL-4 has been shown to activate PPARγ by stimulating production of ligands through the lipoxygenase pathway. It has been suggested that PPARγ may inhibit expression of MMPs by competing with transcription factor AP-1 for binding to a putative composite binding element in the promoters. The objective of this study was to determine whether the suppressive effects of IL-4 on the IL-1 induced expression of MMP-3 involve activation of lipoxygenase and/or PPARγ.

**Results:**

Western blotting revealed the presence of PPARγ in nuclear extract of HGF. IL-1 induced binding of nuclear extract to the putative composite PPRE/AP-1 site was diminished in the presence of pioglitazone, but there was no evidence of any change in the composition of the retarded complexes, and no evidence of PPARγ binding to this site. Nordihydroguaiaretic acid (NDGA), a non-selective lipoxygenase inhibitor, and MK886, a specific inhibitor of 5-lipoxygenase, induced MMP-3 expression synergistically with IL-1. However IL-4 was still able to inhibit MMP-3 expression in the presence of NDGA or MK886 and IL-1. Activation of PPARγ with pioglitazone not only failed to inhibit IL-1 induced expression of MMP-3 mRNA, but rather super-induced MMP-3 in the presence of IL-1. PPARγ antagonist GW9662 failed to abolish the suppressive effects of IL-4. Another PPARγ activator, 15-deoxy-Delta12,14prostaglandin J2 (15dPGJ2), also super-induced MMP-3 mRNA, and this was due at least in part to increased transcription.

**Conclusion:**

IL-4 suppression of IL-1-induced MMP-3 expression in HGF is independent of lipoxygenase activity and activation of PPARγ. Super-induction of MMP-3 by pioglitazone may have important implications for patients using pioglitazone to treat type II diabetes in the presence of chronic inflammation.

## Background

MMP-3 (stromelysin-1) is a metalloproteinase with broad substrate specificity, degrading proteoglycan, laminin, fibronectin, and the non-fibrillar collagens [1]. Perhaps equally important, it is also capable of activating other pro-MMP's, including MMP-1, -8, -9 and -13 [2-4], of inactivating plasminogen activator inhibitor I [5], and of cleaving E-cadherin [6] and FasL [7]. MMP-3 is produced by gingival and synovial fibroblasts, chondrocytes, macrophages, neutrophils, and endothelial cells in response to inflammatory cytokines and mitogens. In periodontitis, MMP-3 is present at increased levels in active disease sites compared to inactive or healthy sites [8-11], and the levels are correlated with clinical parameters and associated with progression of the disease [11]. A similar situation exists in rheumatoid arthritis [12]. Studies of the effects of a promoter polymorphism that affects protein expression has shown that individuals with the high expressing 5A/5A genotype are at increased risk for aneurysms and myocardial infarctions [13-15], while those with the lower expressing 6A/6A genotype show a faster progression of atherosclerosis [15,16]. Taken together, these studies suggest that regulation of MMP-3 levels is important for the maintenance of health, and that either too much or too little can have detrimental effects.

Chronic inflammation in rheumatoid arthritis and periodontitis is characterized by increased levels of IL-1β, TNFα and prostaglandin E2 (PGE2), which contribute to destruction of joints and alveolar bone in part through increased production of matrix metalloproteinases (MMPs) [17-20]. In addition, there is a relative absence of IL-4-producing T cells at sites of inflammation [12,21-25]. This imbalance is progressive, with decreasing levels of IL-4 correlated with loss of collagen and with increasing clinical severity [26]. In addition, polymorphisms in the IL-4 promoter and intron that are associated with decreased serum levels of IL-4 are also associated with increased susceptibility to early onset periodontitis [27]. It has been suggested that correcting this cytokine imbalance in chronic inflammatory conditions might be therapeutic. In fact, adenoviral transfer of IL-4 has been shown to be protective against cartilage degradation induced by injection of rheumatoid arthritis synovial tissue into joints of SCID mice [28] and against collagen-induced arthritis [29].

IL-4 has been shown to inhibit the IL-1 induction of MMP-3 expression in human conjunctival fibroblasts [30], skin fibroblasts [31] and articular chondrocytes [32,33], as well as in synovial fibroblast cultures established from patients with osteoarthritis [34] and gingival fibroblast (HGF) cultures established from patients with periodontitis [35]. The mechanism of the inhibition of MMP-3 by IL-4 in HGF was not determined, although PGE2 was not involved, and DNA binding of transcription factors AP-1 and NF-κB to their consensus sequences was not inhibited [35].

Peroxisome proliferator-activated receptor-γ (PPARγ) is a member of the nuclear receptor super-family. It plays an important role in adipogenesis and insulin sensitization and has also been shown to have a number of anti-inflammatory effects [36]. IL-4 has been shown to activate PPARγ in macrophages by increasing the activity of 15-lipoxygenase, leading to increased production of ligands [37-39]. PPARγ ligands and agonists have been shown to inhibit production of MMP-3 [40,41], and to prevent MMP-mediated tissue destruction in animal models of inflammation, including arthritis [42,43] and periodontitis [44].

A recent study by Francois, *et al*. [45] identified a composite element in the rabbit MMP-1 promoter that exhibited mutually exclusive binding of PPARγ or AP-1 and was responsible for reduced gene expression in the presence of PPARγ agonist rosiglitazone in chondrocytes. The authors reported that computer-based analysis of the human MMP-1 and MMP-3 promoter sequences revealed similar putative composite elements overlapping the well-studied AP-1 sites in each gene. The present study was undertaken to determine whether there is a functional composite PPRE/AP-1 site in the MMP-3 promoter, and if so, whether it is involved in IL-4 suppression of IL-1 induced expression of MMP-3 in HGF. Results show that there is no binding of PPARγ to this site in HGF, and that the suppressive effects of IL-4 are independent of lipoxygenase activity and PPARγ. Surprisingly, a 5-lipoxygenase inhibitor and a PPARγ activator both super-induce MMP-3 in the presence of IL-1.

## Results

### PPAR-γ is present in HGF nuclear extract

**Figure 1:**
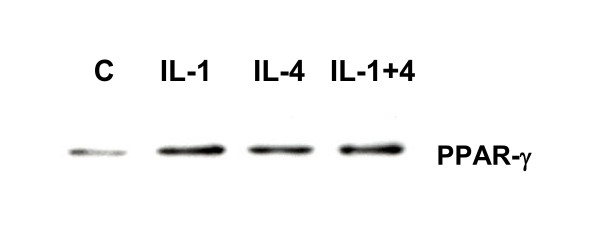
**PPARγ is present in HGF nuclear extract**. Fifteen μg nuclear extract isolated from untreated HGF and HGF treated for 24 hour with IL-1 (10 ng/ml), IL-4 (10 ng/ml), or IL-1 and IL-4 were separated on 10% SDS-PAGE, and transferred to nylon membrane. Proteins were detected with anti-PPAR-γ antibodies followed by chemiluminescence. C = control, no cytokines.

PPARγ is present in the cytosol until ligand binding, at which point it forms heterodimers with retinoid X receptor (RXR) and migrates to the nucleus. In order to demonstrate the presence of PPARγ in HGF, nuclear extract were isolated from control cultures and from HGF cultures treated for 24 hours in the presence of IL-1 (10 ng/ml) and/or IL-4 (10 ng/ml). Results (Figure 1) showed that PPARγ is present in the nucleus of cytokine-stimulated HGF.

### Pioglitazone and IL-4 reduce AP-1 binding to the putative PPRE/AP-1 site, but do not change the composition of the complexes

**Figure 2:**
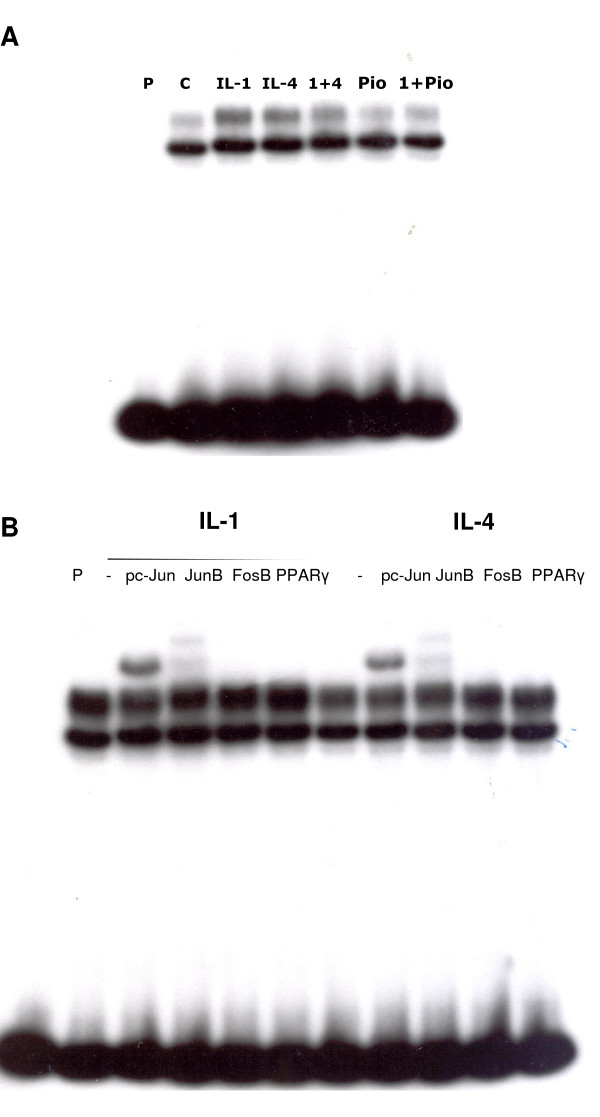
**There is no qualitative change in binding to the PPRE/AP-1 site in the presence of IL-4 or pioglitazone**. A) HGF were incubated in the presence of IL-1 (10 ng/ml), IL-4 (10 ng/ml), and/or pioglitazone (1 μM) as indicated for 1 hour. Five μg nuclear extract were incubated with a ^32^P-labeled oligodeoxynucleotide probe containing the putative composite PPRE/AP-1 site from the MMP-3 promoter. P = probe alone, no extract; C = control, no cytokines. B) HFG were incubated in the presence of IL-1 (10 ng/ml) or IL-4 (10 ng/ml for 1 hour. Five μg nuclear extract were incubated with the PPRE/AP-1 site probe in the presence of the indicated antibodies. P = probe, no extract.

Previous studies have shown that IL-4 inhibition of IL-1 induced expression of MMP-3 is not associated with any alteration in AP-1 binding. However, those studies were done with an AP-1 consensus probe or a probe containing only the AP-1 site from the MMP-3 promoter, neither of which would have encompassed the putative composite PPRE/AP-1 site proposed by Francois et al. [45]. In order to determine whether the composite site is functional in HGF, and whether it is involved in the ability of IL-4 to suppress MMP-3 expression, gelshift assays were performed using an oligonucleotide probe containing the putative composite site. As shown in Figure 2A, IL-1 induced binding to this site was diminished slightly in the presence of IL-4 or pioglitazone. However, neither pioglitazone nor IL-4 alone altered binding patterns compared to control – there were no additional bands or alterations in migration to suggest binding of alternative proteins. PPARγ anti-sera failed to shift the complex, and the ability of anti-sera directed against members of the AP-1 family to super-shift the complex was unchanged in the presence of IL-4 or pioglitazone (Figure 2B). In addition, an oligonucleotide probe corresponding to the consensus PPAR binding site failed to compete for binding to the PPRE/AP-1 probe (data not shown).

### Inhibition of lipoxygenase fails to inhibit MMP-3 expression

**Figure 3:**
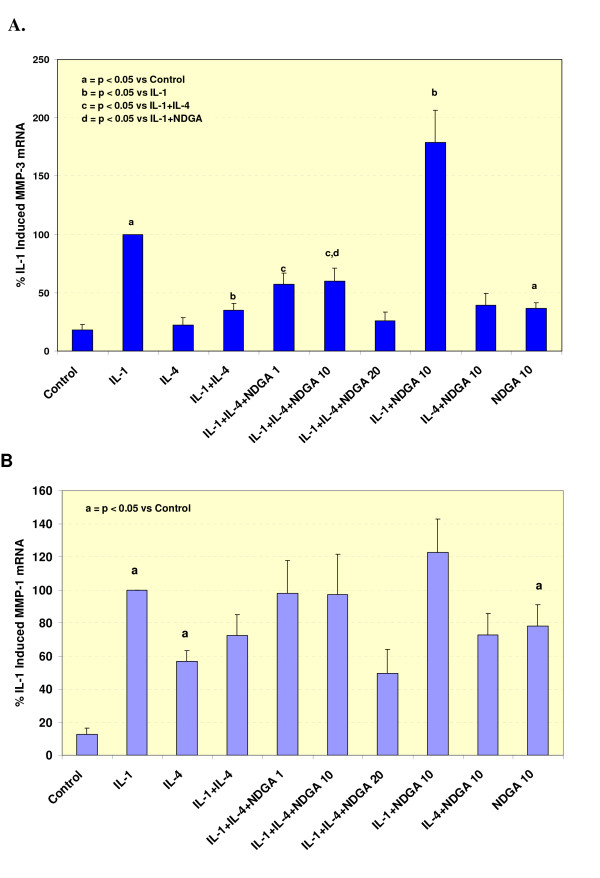
**IL-4 suppression of MMP-3 is independent of lipoxygenase activity, but inhibition of lipoxygenase super-induces MMP mRNA**. HGF cultures were pre-treated with NDGA at the indicated doses for 1 hour prior to addition of IL-1 (10 ng/ml) and/or IL-4 (10 ng/ml). Total RNA was harvested 12 hours after cytokine treatment, quantitated by real-time PCR and normalized to levels of GAPDH. A) MMP-3. The graph represents data from seven independent experiments performed in triplicate, expressed as mean +/- SEM. B) MMP-1. The graph represents five independent experiments performed in triplicate, expressed as mean +/- SEM. Statistical significance was determined using Student's t-test.

In order to determine whether the ability of IL-4 to inhibit IL-1 induced expression of MMP-3 was linked with its ability to induce and/or activate lipoxygenase enzymes, a non-specific lipoxygenase inhibitor, NDGA, was added to the cultures. As shown in Figure 3A, addition of NDGA did result in a dose dependent increase in MMP-3 mRNA in the presence of IL-1 plus IL-4. However, NDGA alone increased MMP-3 expression relative to control. NDGA also resulted in a further induction of MMP-3 in the presence of IL-1. Importantly, IL-4 was still able to inhibit MMP-3 expression induced by IL-1 plus NDGA. Previous results [35] showed that IL-4 fails to inhibit IL-1 induced expression of MMP-1 in HGF. Those results are confirmed here (Figure 3B). IL-4 increased MMP-1 expression compared to control, and there was no significant difference between IL-1 alone and IL-1 plus IL-4. NDGA had no significant effect in the presence of IL-1 and/or IL-4, although it did increase the basal levels of MMP-1 similarly to MMP-3.

**Figure 4:**
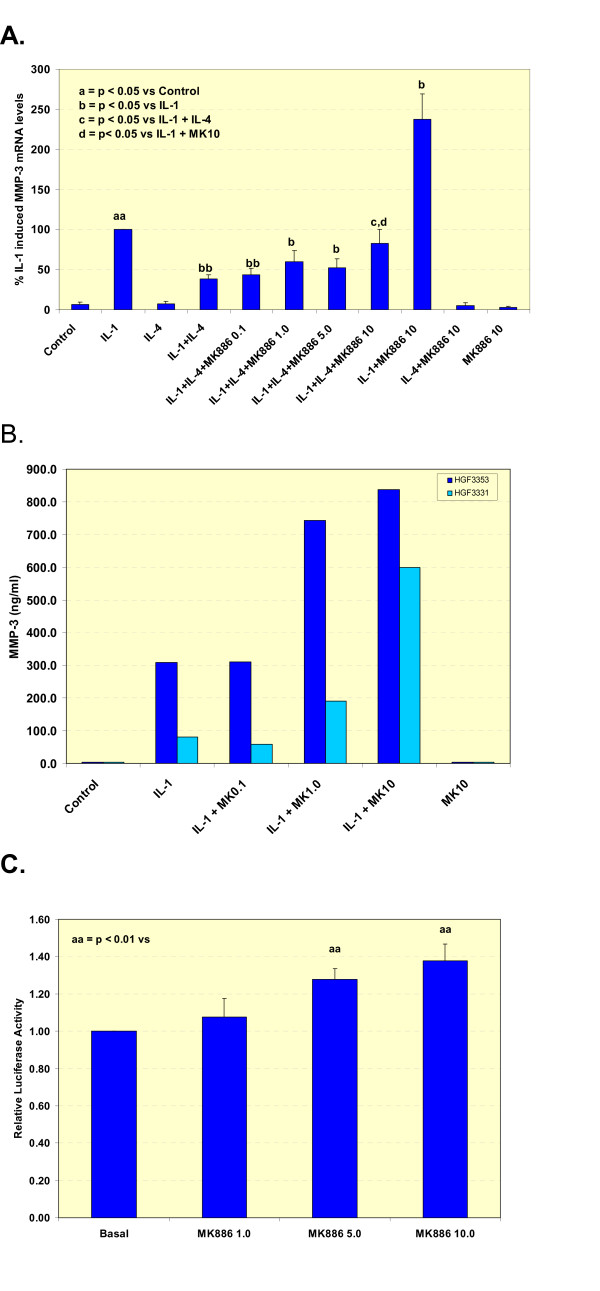
**A specific inhibitor of 5-lipoxygenase, MK886, super-induces MMP-3 mRNA and protein**. HGF cultures were pre-treated with NDGA at the indicated doses for 1 hour prior to addition of IL-1 (10 ng/ml) and/or IL-4 (10 ng/ml). A) Total RNA was harvested 12 hours after cytokine treatment, quantitated by real-time PCR and normalized to levels of GAPDH mRNA. The graph represents five independent experiments performed in triplicate, expressed as mean +/- SEM. B) Conditioned medium was collected from cultures after 24 hours incubation with cytokines +/- MK886 at the indicated doses. MMP-3 protein levels were determined by ELISA. The graph shows data from two different HGF cultures derived from two different donors. C) COS-1 cells were transiently transfected with a luciferase reporter construct containing 2300 bp of the human MMP-3 5' flanking sequence, along with an SV-β-gal reporter as a control for transfection efficiency. MK886 was added 16 hours after transfection, and cells were harvested 24 hours later. Shown are data from three independent experiments performed in triplicate, normalized to levels of β-galactosidase, and expressed relative to control, mean +/- SEM. Statistical significance was determined using Student's t-test.

Similar results were seen with a specific inhibitor of 5-lipoxygenase, MK886, which also increased MMP-3 protein levels (Figure 4). Again, IL-4 was still able to inhibit MMP-3 mRNA induced by IL-1 + MK886. The induction appears to occur at least in part at the transcriptional level, since MK886 increased transcription of an MMP-3 promoter construct in transiently transfected COS cells (Figure 4C).

### Activation of PPARγ with Pioglitazone or 15dPGJ2 does not inhibit IL-1 induced expression of MMP-3 mRNA in HGF

**Figure 5:**
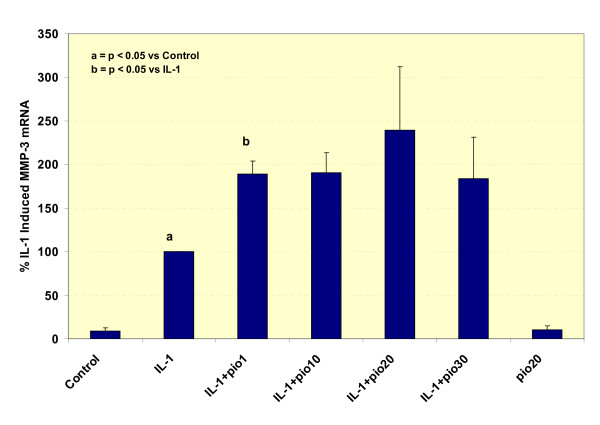
**Pioglitazone fails to inhibit IL-1 induced MMP-3**. HGF cultures were pre-treated with pioglitazone at the indicated doses for one hour before addition of IL-1 (10 ng/ml). Total RNA was isolated at 6 hours, quantitated by real-time PCR and normalized to levels of GAPDH. The graph represents data from three independent experiments performed in triplicate, mean +/- SEM. Statistical significance was determined by Students T-test, p = 0.025 at 1 μM, 0.06 at 10 μM vs IL-1 alone.

**Figure 6:**
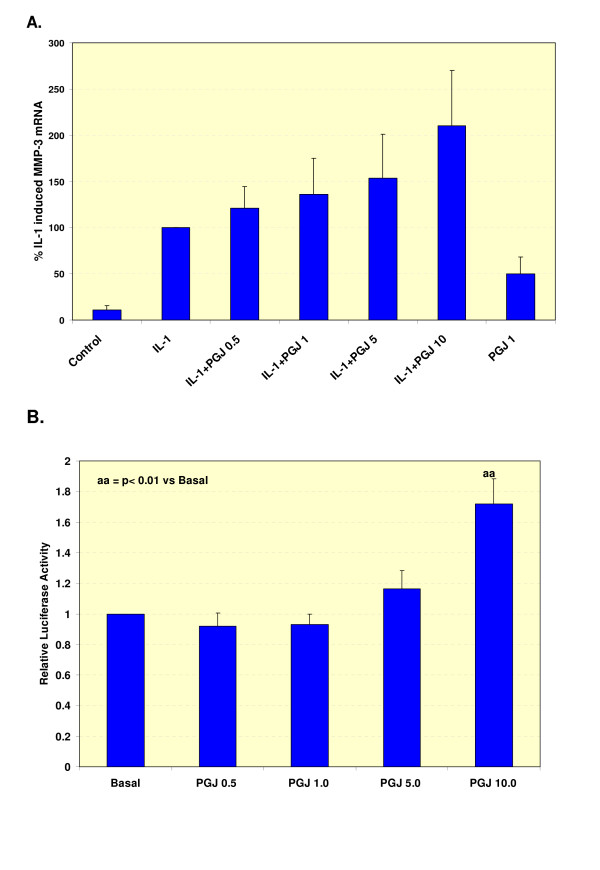
**PPARγ ligand 15dPGJ2 also induces MMP-3 transcription in COS-1 cells**. A) HGF cultures were pre-treated with 15dPGJ2 at the indicated doses for one hour before addition of IL-1 (10 ng/ml) and/or IL-4 (10 ng/ml). Total RNA was isolated at 12 hours, quantitated by real-time PCR and normalized to levels of GAPDH. The graph represents data from six independent experiments performed in triplicate, mean +/- SEM. B) COS-1 cells were transiently transfected with a luciferase reporter construct containing 2300 bp of the human MMP-3 5' flanking sequence, along with an SV-β-gal reporter as a control for transfection efficiency. 15dPGJ2 was added 16 hours after transfection, and cells were harvested 24 hours later. Shown are data from three independent experiments performed in triplicate, normalized to levels of β-galactosidase, and expressed relative to control, mean +/- SEM. Statistical significance was determined using Student's t-test.

**Figure 7:**
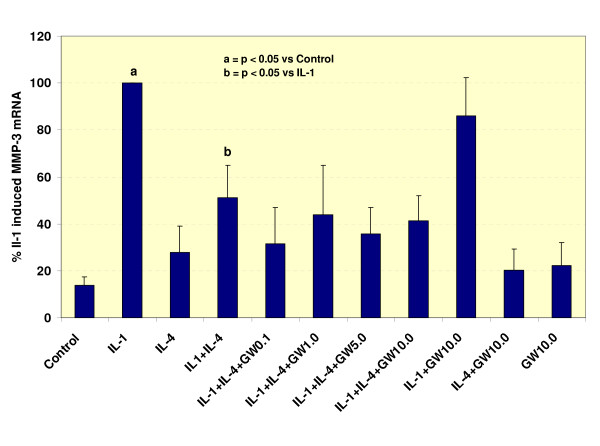
**PPAR antagonist GW9662 fails to reverse the inhibitory effects of IL-4**. HGF cultures were pre-treated with GW9662 at the indicated doses for one hour before addition of IL-1 (10 ng/ml) and/or IL-4 (10 ng/ml). Total RNA was isolated at 12 hours, quantitated by real-time PCR and normalized to levels of GAPDH. The graph represents data from six independent experiments performed in triplicate, mean +/- SEM. Statistical significance was determined by Students T-test.

The fact that IL-4 could still inhibit MMP-3 expression in the presence of NDGA suggested strongly that lipoxygenase activation is not involved in the inhibition, and the gelshift results demonstrate that there is no competition between PPARγ and AP-1 for binding to the promoter. However, it is possible that IL-4 could activate PPARγ through increased production of alternative ligands [39], and that activation of PPARγ might inhibit MMP-3 expression through another mechanism, such as competition for transcriptional co-activators [46,47]. In order to determine whether activation of PPARγ leads to suppression of MMP-3 expression in these cells, HGF cultures were pre-treated with increasing doses of the selective PPARγ agonist pioglitazone for one hour before addition of IL-1. Total RNA was harvested six hours later and analyzed by real-time PCR. Results of three independent experiments (Figure 5) showed that pioglitazone was not able to inhibit IL-1 induced expression of MMP-3. In fact, pioglitazone further increased MMP-3 mRNA in the presence of IL-1 (p = 0.025 at 1 μM and p = 0.060 at 10 μM). Likewise, another activator of PPARγ, 15dPGJ2 also seemed to super-induce MMP-3 mRNA expression in the presence of IL-1, although this did not reach statistical significance (Figure 6A). In transiently transfected COS cells, however, 15dPGJ2 cause a small but significant induction of transcription of an MMP-3 promoter construct (Figure 6B). In accordance with these findings, the ability of IL-4 to inhibit MMP-3 expression was not affected by the PPARγ antagonist, GW9662 (Figure 7).

## Discussion

Results shown here demonstrate that there is no competition between PPARγ and AP-1 for binding to the MMP-3 promoter, and that IL-4 suppression of IL-1-induced expression of MMP-3 in HGF does not involve activation of lipoxygenase enzymes and/or PPARγ. However, the fact that inhibitors of lipoxygenase and an activator of PPARγ both resulted in super-induction of MMP-3 in the presence of IL-1 suggests that these factors may play a role in MMP-3 gene regulation.

Previous studies with the AP-1 site failed to detect any changes in DNA binding in cells treated with IL-1 plus IL-4 as compared to IL-1 alone [34,35]. Use of a larger probe containing the putative PPRE/AP-1 composite site did show decreased intensity of retarded bands in the presence of IL-4 (Figure 2). However there was no change in the migration of the retarded bands as one would expect if PPARγ were binding to the site in competition with AP-1, and a PPARγ specific antibody failed to supershift the bands. IL-4 has been reported to stimulate expression of JunB in peripheral blood monocytes from patients with atopic dermatitis [48]. Since JunB is less transcriptionally active than c-Jun [49,50], such a shift in the composition of AP-1 dimers would be expected to decrease transcription of at least some AP-1 dependent genes. Use of antisera against phosphorylated (active) c-Jun and Jun B suggest that IL-4 is not interfering with activation of c-Jun, nor is it changing AP-1 activity by causing the formation of less active dimers containing JunB. Taken together, these data demonstrate that IL-4 inhibition of MMP-3 expression cannot be explained by competition for binding to the AP-1 site or by changes in the composition of the AP-1 dimer.

Activation of PPARγ has been shown to inhibit cytokine stimulated gene expression in a number of ways, including inhibition of NF-κB and AP-1 activity through competition for limiting amounts of transcriptional coactivators [46,47]. Furthermore, IL-4 has been shown to activate PPARγ by stimulating the production of ligands through the lipoxygenase pathway [37-39] and in at least one case, through the production of alternative ligands [39].

The fact that IL-4 could still inhibit MMP-3 expression in the presence of NDGA or MK886 suggests that the mechanism of IL-4 inhibition does not require activation of lipoxygenase enzymes. Super-induction of IL-1 stimulated MMP-3 expression by NDGA was surprising in light of the fact that NDGA is known to inhibit AP-1 activity [51] and it has been shown to inhibit expression of MMP-3 in response to IL-17 [52]. Since pioglitazone also resulted in stimulation of MMP-3 expression while inhibiting AP-1 binding (Figures 2, 5), it is clear that other pathways are more important than AP-1 in this context.

As reported earlier [35], IL-4 failed to inhibit IL-1 induction of MMP-1. Although NDGA induced MMP-1 over control levels, it failed to super-induce MMP-1 with IL-1 as it did with MMP-3. These results represent another addition to a growing list of situations in which these two genes are not co-ordinately regulated. In one instance, Otani et al [53] showed induction of MMP-3, but not MMP-1 in the presence of cycloheximide. They suggested that cycloheximide might be inducing MMP-3 expression by preventing translation of a "labile repressor". It is interesting to note as well that the increased transcription of the MMP-3 promoter construct was observed only with the construct containing the 5A polymorphism and not in the construct containing the 6A polymorphism (data not shown). Since this polymorphic site has been shown to act as a repressor element in normal fibroblasts [16,54], it is tempting to speculate that these reagents are affecting the binding or activity of transcription factor(s) binding to this site in order to relieve the repression. Further experiments are needed to address these questions.

Interactions between the cyclooxygenase, lipoxygenase and PPAR pathways are overlapping and complex. Arachidonic acid is a common substrate for both cyclooxygenase and lipoxygenase enzymes. Expression of 15- and 5-lipoxygenase enzymes is often reciprocal [55,56]. Activation of PPARγ by troglitazone has been shown to inhibit expression of 5-lipoxygenase in rheumatoid arthritis synovial fibroblasts [57], lipoxygenase inhibitors can activate PPARγ in breast cancer cells [58] and COX-2 expression in macrophages has been postulated to be regulated by negative feedback mediated by PPARγ [59].

One potential explanation for the unexpected effects of NDGA and MK886, therefore, is that inhibition of lipoxygenase enzymes leads to an excess of arachidonic acid, which can then be converted to prostaglandins by cyclooxygenases. If pioglitazone and/or 15d PGJ2 caused a down-regulation of 5-lipoxygenase expression and/or activity, then they too could result in increased levels of arachidonic acid and increased production of prostaglandins.

This is not straight-forward however, since the effects of PGE2 on MMP-3 expression are variable, ranging from induction [60,61], to inhibition [62-64], to no effect [30,65]. A particularly interesting study by Rowanpura et al. [66], showed that the effects of PGE2 on IL-1 stimulated expression of MMP-3 differ in HGF derived from healthy tissue as opposed to HGF derived from patients with severe periodontitis. In HGF from healthy donors, PGE2 inhibited the IL-1 induction of MMP-3, whereas in diseased tissue, it resulted in further stimulation. This difference was shown to result from a change in PGE2 receptors, from EP2 and EP4 in healthy tissue to predominantly EP1 in diseased tissue.

Since our HGF were derived from patients with periodontitis, it is possible that addition of NDGA, MK886 and/or pioglitazxone resulted indirectly in increased production of PGE2, which caused super-induction of MMP-3 as described by Ruwanpura, et al. [66]. It is important to note that in previous studies using similarly derived HGF, addition of exogenous PGE2 failed to abrogate the suppressive effects of IL-4 on MMP-3 expression [35], but the effects of exogenous PGE2 alone or together with IL-1 were not tested. In the present study, there was some variation in the magnitude of responses, but the responses themselves were qualitatively consistent among cultures established from different individuals (n = 7 for NDGA, n = 5 for MK886, n = 3 for pioglitazone, n = 6 for 15d PGJ2 experiments).

Aside from demonstrating that PPARγ is not involved in the effects of IL-4 on MMP-3 expression, the failure of pioglitazone and 15dPGJ2 to inhibit MMP-3 expression (Figures 5 and 6) was surprising because other studies have shown inhibition of MMP-3 expression by activators of PPARγ [40,41]. The most obvious explanation for the super-induction would be the presence of a PPRE in the MMP-3 promoter. Computer analysis of the promoter sequence [Genomatix, [67]] failed to identify any PPRE. However, a recent study found that of the 73 known functional PPRE, only 2 conformed to the DR1 consensus, and 8% of them had 5 or more mismatches [68]. Therefore, this possibility cannot be excluded without further study.

The possibility of PPARγ-independent effects of pioglitazone and 15dPGJ2 also cannot be ruled out. 15dPGJ2 has been shown to have several effects independent of PPAR, including inhibition of NFκB [69]. Pioglitazone has been shown to up-regulate AMP-activated kinase in rat muscle and in NIH3T3 cells)[70] and to increase LPS stimulated production of PGE2 at high doses [71]. Aizawa et al. [72] showed that pioglitazone increased the IL-1 induced expression of inducible nitric oxide synthase (iNOS) in vascular smooth muscle cells (VSMCs) independent of PPARγ.

Both NDGA and pioglitazone have anti-oxidant properties which have been linked to their ability to inhibit the activity of redox sensitive transcription factor NF-κB [43,73,74]. NF-κB has been implicated in several studies to be involved in up-regulation of MMP-3 in inflammation [75-77], but more recent studies have shown that NF-κB binds to a polymorphic repressor element in the MMP-3 promoter in competition with zinc-binding protein 89 (ZBP-89) to limit IL-1 induced transcription [54], unpublished]. Further study is needed to determine more precisely the direct and indirect roles of NF-κB in MMP-3 regulation and how these roles are affected by any alterations in eicosanoid synthesis or actions that might take place in the context of chronic inflammation.

## Conclusion

In summary, these data demonstrate that the ability of IL-4 to suppress IL-1 induced expression of MMP-3 does not involve activation of PPARγ and/or lipoxygenase activity in HGF. However, the fact that lipoxygenase inhibition and PPARγ activation were both able to super-induce MMP-3 mRNA suggests that these pathways are involved in MMP-3 gene regulation. These results also suggest that treatment with drugs that target these pathways in the context of chronic inflammation could have some unpredicted effects. Further study is necessary to determine how these effects are mediated and to what extent they are relevant to the disease process(es).

## Methods

### Cell culture

Human gingival tissue from patients undergoing periodontal surgery was obtained from Howard M. Sobel, D.D.S. and Kevan S. Green, D.M.D. of Sobel Periodontal Associates, P.C. The tissue was processed by enzymatic dispersion to produce primary cultures. Cells were maintained in Eagle's Minimal Essential Medium (EMEM) supplemented with 10% fetal bovine serum and antibiotic/antimycotic (penicillin, streptomycin, amphotericin; Gibco BRL, Grand Island, NY). Cells between passages 3 and 5 were used for experiments. COS-1 cells were obtained from ATCC and maintained as suggested.

### RNA isolation and real-time PCR

Cells were serum-deprived for 16 hours in serum-free EMEM supplemented with 10% ITS (insulin, transferrin, sodium selenite; Sigma, St. Louis, MO) prior to the addition of NDGA, MK886, pioglitazone, 15dPGJ2 or GW9962 (Sigma) in the indicated doses. After one hour incubation, 10 ng IL-1β/ml (DuPont-Merck Corp., Wilmington, DE) was added, in the presence or absence of 10 ng/ml IL-4 (Gibco BRL, Grand Island, NY). Total RNA was isolated 6 or 12 hours later using the RNAqueous-4PCR kit (Ambion, Austin, TX) according to manufacture's instructions and reverse transcribed into cDNA using the High Capacity cDNA Archive Kit (Applied Biosystem, Foster City, CA) and Thermal Cycler Genius. Real Time PCR reactions were consisted of 2.5 μl of cDNA, 12.5 μl of Taqman Universal PCR master mix (Applied Biosystem), 8.75 μl H2O and 1.25 μl probes (stromylsin-MMP-3, collagenase I-MMP-1, Glyceraldehyde-3-Phosphate Dehydrogenase-GAPDH; Applied Biosystem) or 2 μl of cDNA, 6.8 μl of H2O, 10 μl of the Premix *Ex Taq*™ (master mix and 0.4 μl ROX Reference Dye; TAKARA, Madison, WI) and 0.8 μl probes. Real Time PCR analysis was done using the Applied Biosystem 7500 Real Time PCR system. Reactions were done in triplicate and results were normalized to GAPDH.

### Nuclear extract isolation and gelshift assays

Nuclear extracts were isolated according to the method of Schreiber et al. [78] and quantified in mini-Bradford assays (Pierce, Rockford, IL). Synthetic oligonucleotides corresponding to the putative composite PPRE/AP-1 site (5' GAAAGCAAGGTGAGTCAAGCTG 3') [45] were labelled by T4 polynucleotide kinase in the presence of γ^32^P-ATP. Binding reactions contained 5 μg protein, 20 mM Hepes-OH pH 7, 50 mM NaCl, 0.2 M EDTA, 5% glycerol, 4 μg dIdC and 10,000 cpm probe.

#### Western Blotting

Fifteen μg nuclear extract were separated on 10% SDS-polyacrylamide gels. Proteins were transferred to Immobilon-P Transfer Membrane (Millipore) and the membrane was blocked overnight with Super Block blocking buffer (Pierce). The blot was then incubated with anti-PPAR-γ antibody (Santa Cruz) diluted 1:1000 in Super Block for one hour at room temperature. After washing three times in TBST (20 mM Tris-HCl pH 7.5, 150 mM NaCl, 0.5% Tween 20), the blots were incubated with a goat anti-rabbit secondary antibody conjugated to horseradish peroxidase (Pierce) for one hour. After three more washes in TBST, the blot was incubated with SuperSignal West Pico Chemiluminescent Substrate (Pierce) and exposed to X-ray film.

### Transient transfection

A luciferase reporter construct containing 2300 bp of the MMP-3 5' flanking region and the 5T promoter polymorphism has been described previously [79]. Transient transfections were performed in triplicate using Lipofectamine Plus (GibcoBRL), with SV-βgal as a control for transfection efficiency. Cells were harvested 36 hours later.

### Determination of MMP-3 protein levels

MP-3 protein levels were quantitated by ELISA (Calbiochem, Darmstadt, Germany) in conditioned media of human gingival fibroblasts (HGF) that were untreated, treated with 10 ng IL-1/ml alone or treated with both 10 ng IL-1/ml and 0.1, 1.0 or 10 μM MK886 for 24 hours.

## Authors' contributions

DS participated in drafting the manuscript and performed real-time PCR experiments with NDGA and pioglitazone; MJ performed the Western blot and EMSA, as well as real-time PCR and transfection with 15dPGJ2; MC and CG completed the real-time PCR experiments with MK886 and GW9662. MC did the MMP-3 ELISA and MK886 transfection experiments. GG participated in analysis of real-time PCR data. RCB conceived the experiments, analyzed the results and completed the manuscript. All authors approved the manuscript.
